# IFN-γ restores the impaired function of RNase L and induces mitochondria-mediated apoptosis in lung cancer

**DOI:** 10.1038/s41419-019-1902-9

**Published:** 2019-09-09

**Authors:** Huijing Yin, Zhengyu Jiang, Shuoer Wang, Ping Zhang

**Affiliations:** 1 Cancer Institute, Fudan University Shanghai Cancer Center, 200032 Shanghai, China; 2 Department of Oncology, Shanghai Medical School, Fudan University, 200032 Shanghai, China; 3 Department of Immunology, Tongji University School of Medicine, 1239 Siping Road, 200092 Shanghai, China; 4 Faculty of Anesthesiology, Changhai Hospital, Second Military Medical University/Naval Medical University, 200433 Shanghai, China; 5 Central Laboratory, The Fifth People’s Hospital of Shanghai Fudan University, Shanghai, China

**Keywords:** Lung cancer, Apoptosis

## Abstract

RNase L is an essential component in interferon (IFN)-mediated antiviral signaling that showed antitumor effects in cancer. Cancer immunotherapy based on interferon has achieved encouraging results that indicate an applicable potential for cancer therapy. Here we showed that function of RNase L, though highly upregulated, was functionally impaired both in nuclear and cytoplasm in lung cancer cells. In normal lung epithelial cells, RNase L activation induced by 2–5A promoted nuclear condensation, DNA cleavage, and cell apoptosis, while in lung cancer cells, these processes were inhibited and RNase L-mediated downregulation of fibrillarin, Topo I and hnRNP A1 was also impaired in lung cancer cells. Moreover, the impairment of RNase L in lung cancer cells was due to the elevated expression of RLI. Application of IFN-γ to lung cancer cells led to enhanced expression of RNase L that compensated the RLI inhibition and restored the cytoplasmic and nuclear function of RNase L, leading to apoptosis of lung cancer cells. Thus, the present study discovered the impaired function and mechanism of RNase L in lung cancer cells and proved the efficacy of IFN-γ in restoring RNase L function and inducing apoptosis in the lung cancer cell. These results indicated the RNase L as a therapeutic target in lung cancer cells and immunotherapy of IFN-γ may serve as an adjuvant to enhance the efficacy.

## Introduction

Research and treatment for lung cancer have focused more on the altered intrinsic mechanism that results in tumorigenesis^1,2^. For example, abnormal expression and function of the molecule that regulates transcription or translation becomes a promising target for cancer treatment^3^. In-depth knowledge of such abnormal molecule and mechanism, as well as targeted correction of the abnormality, may help suppress the cancerous phenotype or inducing apoptosis that attenuates the progression of lung cancer cells.

RNase L is an essential component of the 2–5A/RNase L system in interferon (IFN)-mediated antiviral signaling^4,5^, which mainly targets the RNA substrate to initiate cellular defense against the virus. The IFN initiate the activation of Janus-activated kinase (JAK)-signal transducer and activator of transcription factor (STAT) signaling and induce the expression of oligoadenylate synthase (OAS)^1^. Intracellular double-stranded RNA activated OAS and synthesized 2–5A from ATP. The latter further activates RNase L as endoribonuclease to catalyze single-stranded RNA (ssRNA) or rRNA, inhibiting virus replication and inducing cell apoptosis^6–10^. Recently, the broader function of RNase L has also been identified in regulating RNA metabolism, proliferation, apoptosis, differentiation, and autophagy^3,11,12^. These functions are achieved through the cleavage of rRNA or regulate protein translation through translational termination or direct mRNA catalyzation by RNase L^3,13–15^. Based on these regulatory mechanisms, RNase L is also identified as an antitumor effector, which inhibits c-Myc expression through suppressing hnRNP A1-mediated stabilization of mRNA^14,16,17^. RNase L could also attenuate cancer progression through suppressing matrix metalloproteinase activity, while a deficiency in RNase L promotes carcinogenesis^3^. However, RNase L is strictly regulated by the activation of 2–5A as well as the suppression from RNase L inhibitor, RLI^7,18^. It remains largely unknown about how RNase L expresses and functions in cancer cells and whether targeting RLI/RNase L or promoting RNase L activity would affect the cancerous phenotype and helps to suppress cancer progression.

Cancer immunotherapy based on IFN has attracted much attention for its encouraging results from melanoma, colon cancer, hepatoma, and hematological malignancies^1^. Researches have demonstrated the antitumor activity of type I and type III IFN through several mechanisms, including anti-angiogenesis, anti-proliferation, and inducing cell cycle arrest and apoptosis^1,2,14,19^. However, the effects of type II IFN on cancer remains largely unknown. IFN-γ could interact with its receptor formed by IFNGR1 and IFNGR2, leading to the activation of JAK1 and JAK2, and subsequently inducing STAT1 dimerization and gene transcription^1^. It remains unknown whether IFN-γ could affect the cancerous phenotype or inducing apoptosis of cancer cells through RNase L. In the present study, we reported the abnormal expression, impaired function, and regulatory mechanism of RNase L in lung cancer cells. We also demonstrated the apoptosis-induction effects of IFN-γ in lung cancer cells through a unique compensatory mechanism in restoring the RNase L function.

## Materials and methods

### **C**ell culture and reagents

Human lung cancer cell, NCI-H157 (non-small cell lung adenocarcinoma cells), GLC-82 (Chinese lung adenocarcinoma cells), Calu-3 (lung adenocarcinoma cells derived from malignant pleural effusion), LTEP-s (lung squamous carcinoma cells), NCI-H520 (non-small cell lung squamous carcinoma cells), NCI-H209 (small cell lung cancer cells), PG-LH7 (pulmonary giant cell carcinoma cells), T47D (human breast carcinoma cells), and BEAS-2B (human normal lung epithelial cells) were kindly provided by Stem Cell Bank, Chinese Academy of Sciences. Cells were cultured in Dulbecco’s modified Eagle’s medium or 1640 culture medium (Hyclone, USA) supplemented with 10% fetal bovine serum (Gibco, USA) and 1% Penicillin–Streptomycin (Hyclone, USA).

### Extraction of cell lysate, the cytosolic, mitochondrial, and nuclear protein

The cell lysate was extracted by RIPA (Cell Signaling Technology, CST, USA) according to the manufacturer’s protocol. Protein in the cytoplasm and nucleus was extracted by using NE-PER Nuclear and Cytoplasmic Extraction (Pierce, USA). Briefly, pre-cooling CRE I was added into centrifuged cells and cooled on ice for 10 min. Then cells were vibrated for 15 s and centrifuged at 4 °C, 14,000 × *g* for 5 min. Cytoplasmic protein in the supernatant was collected. Residual sediment was added with 100 μl pre-cooling NER and vibrated for 15 s. After three times of 10-min cooling, and 15-s vibration and centrifuged at 4 °C, 14,000 × *g* for 10 min, nuclear protein in the supernatant was collected. Mitochondrial protein was extracted according to the manufacturer’s protocol of Mitochondrial/Cytoplasmic Component Extraction Kit (Millipore, USA). The extracted protein was then subjected to quantification by using a BCA kit (Thermo, USA) and 20 μg protein was used for sodium dodecyl sulfate-polyacrylamide gel electrophoresis (SDS-PAGE).

### Western blot (WB) and immunoprecipitation

The protein was separated on SDS-PAGE, transferred to polyvinylidene difluoride membranes (Millipore, USA), and blocked with 5% non-fat dry milk in TBST. After three times of washing with TBST, following primary antibodies dissolved in antibody buffer (Keygentec, China) were used: anti-human RNase L (sc-74405, Santa Cruz, USA), RLI (ab185548, Abcam, USA), Fibrillarin (#2639, Cell Signaling Technology, USA), Topo I (20705-1-AP, Proteintech, China), hnRNP A1 (#8443, Cell Signaling Technology, USA), Cytochrome C (#4280, Cell Signaling Technology, USA), Prohibitin (10787-1-AP, Proteintech, China), COX IV (#4850, Cell Signaling Technology, USA), Bax (50599-2-Ig, Proteintech, China), Bak (33326-1, SAB biotech, USA), Caspase-9 (#9505, Cell Signaling Technology, USA), Caspase-3 (#9662, Cell Signaling Technology, USA), poly ADP-ribose polymerase (PARP; #5625, Cell Signaling Technology, USA), OAS1 (#14498, Cell Signaling Technology, USA); OAS2 (sc-374238, Santa Cruz), and OAS3 (SAB1300335, Sigma-Aldrich). After the secondary antibody incubation, the membrane was washed three times with TBST and exposed with ECL (Millipore, USA). The corresponding semi-quantitative analysis was performed by measuring the optical density using the ImageJ software.

For co-immunoprecipitation, antibodies used were as follows: anti-human RNase L (sc-74405, Santa Cruz, USA), anti-Bax (#2774, Cell Signaling Technology, USA) and anti-Bak (#3814, Cell Signaling Technology, USA). Briefly, 5 μl antibodies were added to cell lysate (50 μg protein) and incubated at rotator at 4 °C for 4 h. Then 50 μl Protein A/G-Sepharose Beads (Pierce, USA) was added, mixed, and rotated for 4 °C overnight. The beads were centrifuged at 3000 rpm, 4 °C for 20 s. Beads were then washed by phosphate-buffered saline (PBS) for 3 times and centrifuged at 3000 rpm, 4 °C for 20 s to complete a total of three times of washing. Then SDS loading was added and the sample degenerated at 100 °C for 5 min. The sample was centrifuged and subjected to SDS-PAGE and analyzed with the indicated antibodies for WB.

### Immunocytofluorescence (ICF) and immunocytochemistry (ICC)

For ICF, cell slides were fixed with 4% paraformaldehyde at 4 °C for 30 min, permeabilized with 0.1% Triton-100 dissolved in PBS at room temperature for 20 min, and blocked with normal goat serum for 1 h. Mouse anti-RNase L and rabbit anti-fibrillarin (Abcam, USA) were added and incubated at 4 °C overnight. After three times of washing with PBS, rhodamine-conjugated goat anti-mouse IgG or fluorescein isothiocyanate-conjugated goat anti-rabbit IgG (Jackson ImmunoResearch, USA) was added and incubated in room temperature for 1 h. After washing, nuclear was stained with 4,6-diamidino-2-phenylindole (DAPI) for 2 min. Confocal microscopy was performed with a Nikon N1 and images were processed with a cooled CCD camera and NIS Viewer software.

For ICC, cells were fixed with 4% paraformaldehyde at 4 °C for 30 min, washed with PBS, incubated with 0.3% hydrogen peroxide for 20 min, and blocked with normal goat serum for 1 h. Mouse anti-RNase L was added and incubated at 4 °C overnight. After washing with TBS, sections were incubated with biotinylated goat anti-mouse (1:1000, Jackson ImmunoResearch Laboratories) for 30 min within the humid incubator. The signal was detected using the avidin–biotin–peroxidase complex (PK-6100, Vector Laboratories) in combination with DAB substrate (SK-4100, Vector Laboratories) and the sections were washed in TBS-T (pH 7.4). Finally, the sections were rinsed in distilled water, counterstained with hematoxylin (H-3401, Vector Laboratories), and mounted on microscopic sides. Microscopy was performed with a Nikon Eclipse and images were processed with the NIS Viewer software.

### Detection of RNase L activity

This measurement refers to the method reported by Rusch et al.^20^. 2–5A-transfected cells were lysed with TRIzol and then 200 μl chloroform, centrifuged at 12,000 rpm, 4 °C for 10 min, and the supernatant was collected. Then 500 μl isopropanol was added, mixed, cooled on ice for 20 min, and centrifuged at 4 °C, 12,000 rpm for 10 min. The supernatant was discarded and 1 ml 70% ethanol was added for washing. Then 20 μl TMC buffer (10 mM Tris-HCl (pH 7.5), 5 mM MgCl_2_, 100 nM CsCl) was added to reconstitute RNA and immediately subjected to RNA electrophoresis. Cleavage products and 18S and 28S rRNAs were observed under ultraviolet exposure. RNase L activity was measured quantitatively according to the cleavage products of rRNA.

### RNase L dimerization assay

Cells were washed with pre-cooling PBS and then loading buffer (20 mM Tris-HCl (pH 8.0), 137 mM NaCl, 10% glycerol, 1% NP-40, 2 mM EDTA) was added to lysate cells. Then Cocktail buffer (a mixture of protease inhibitor and phospholipase inhibitor; Roche, USA) and cells were incubated on ice for 20 min and centrifuged at 4 °C, 12,000 rpm for 15 min. The supernatant was collected and mixed with 5 mg/ml dimethyl suberimidate (Fluka, USA) and incubated at room temperature for 30 min. Then samples were mixed with isometric SDS loading buffer and subjected to SDS-PAGE for WB.

### 2–5A synthesis and transfection

The synthesis of 2–5A refers to the method reported by Kerr et al.^21^. The reaction system of 2–5A (p3(A2′p)*n* A, *n* = 1 to ≥3) was as follows: OAS1 buffer (20 mM HEPES (pH 7.6), 20 mM Magnesium acetate, 20 mM KCl, 1 mM EDTA), 20 μg/ml 2′, 5′-OAS1 (Abnova, USA), 1 μg/ml poly (I:C) (InvivoGen, USA), and 10 mM ATP (pH7.4) (CST, USA). The mixture was incubated at 37 °C for 24 h, filtered by Centricon-10 (Millipore, USA), and then centrifuged at 3000 × *g* for 15 min. The analysis of 2–5A synthesis was carried using a Mono Q HR 5/5 column (GE Healthcare, USA). A 200 μL aliquot was applied to the column equilibrated in Buffer A (20 mM Tris–HCl, pH 7.5) and fractionated in a linear gradient of 0–20% and 20–22.8% of Buffer B (1 M NaCl, 20 mM Tris–HCl pH = 7.5) using 18 column volumes (CV) and 50 CV, respectively. Concentration of the oligoadenylates were determined by application of small aliquots (~20 μl) to the Mono Q HR 16/10 column. For concentration determination, ATP was used as an internal control. 2–5A was diluted by Opti-MEM (Invitrogen, USA) and transfected by using calcium phosphate coprecipitation.

### Small interfering RNA (siRNA) interference

RNase L siRNA (sc-45965), RLI siRNA (sc-60117), OAS3 siRNA (sc-61245), and control siRNA (sc-37007) were purchased from Santa Cruz and diluted to 10 μM with TE buffer. Lipofectamine® RNAiMAX (Invitrogen, USA) was used for siRNA transfection. Cells were seeded at six-well culture plate overnight (1 × 10^6^/well for WB, 1 × 10^4^/well for ICF) and culture medium was then changed to Opti-MEM. The transfection system was prepared as follows: 9 μl Lipofectamine® RNAiMAX diluted in 150 μl Opti-MEM and then mixed with siRNA-Opti-MEM. After 5 min of mixing, transfection system was then added to wells and interference was carried out for 48 h.

### TUNEL (terminal deoxynucleotidyl transferase-mediated dUTP-fluorescein nick end labeling) analysis

For TUNEL analysis, cell slides were fixed with 1% paraformaldehyde at room temperature for 10 min and then pre-cooled with ethanol/acetic acid (2:1) at −20 °C for 5 min. After PBS washing, slides were incubated with terminal deoxynucleotidyl transferase (TdT) buffer (Millipore, USA) at 37 °C for 1 h and washed with a stop solution for 15 s and PBS for 3 times. Then slides were incubated with rhodamine-conjugated anti-digoxigenin antibody (Millipore, USA) at 37 °C for 30 min, washed with PBS, stained with DAPI for 2 min, and PBS washed. Slides were subjected to microscopy and the images were merged by Image J.

### Apoptotic DNA ladder extraction

Cells were resuspended from culture plates and washed with PBS. Apoptotic DNA ladder was extracted according to the manufacturer’s protocol of Apoptotic DNA Ladder Extraction Kit (BioVision, USA): cells were suspended in 50 μl DNA Ladder extraction buffer and centrifuged at 1600 × *g* for 5 min. Then the supernatant was removed and the previous procedure was repeated with the sediment. The supernatant of two times centrifuge was collected and 5 μl Enzyme A was added and incubated at 37 °C for 10 min. Then 5 μl Enzyme B was added and incubated at 50 °C for 30 min. The mixture was then added with 5 μl ammonium acetate and 100 μl isopropanol and incubated at −20 °C for 10 min. Then the mixture was centrifuged at 14,000 × *g* for 10 min and washed with 500 μl 70% ethanol. DNA was suspended with 30 μl DNA suspension buffer and 15 μl was used for agarose gel electrophoresis.

### Statistical analysis

Data were expressed as the mean ± SEM. Student’s *t* test was conducted for comparisons between two groups, and one-way analysis of variance was performed for comparisons among several groups. A *P* value <0.05 was considered to be statistically significant.

## Results

### RNase L is upregulated with impaired function in lung cell lines

**Fig. 1:**
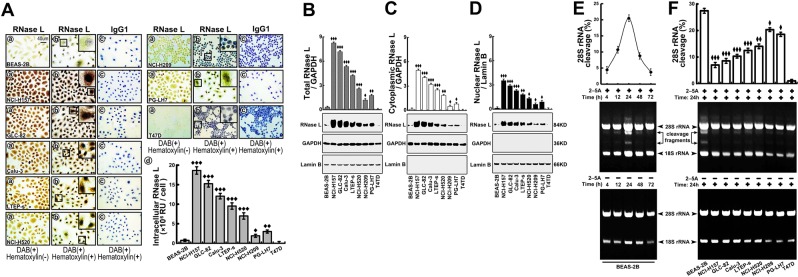
Elevated expression with impaired cleavage activity of RNase L in lung cancer cells. **a** Immunocytochemistry and quantitative analysis of RNase L in normal pulmonary epithelial cells (BEAS-2B), lung cancer cell (NCL-H157, GLC-82, Calu-3, LTEP-s, NCL-H520, NCL-H209, PG-LH7), and breast cancer cell (T47D). **b**–**d** Western blot of the total (**b**), cytoplasmic (**c**), and nuclear (**d**) RNase L in the indicated cell lines. **e** Cleavage activity to 28S rRNA in the indicated time points of RNase L in BEAS-2B cells (*n* = 3). **f**: Cleavage activity to 28S rRNA at 24 h of RNase L in lung cancer cell lines and breast cancer cells (T47D) (*n* = 3). ^♦^*p* < 0.05; ^♦♦^*p* < 0.01; ^♦♦♦^*p* < 0.001, Student’s *t* test

To investigate the expression feature of RNase L in lung cancer cells lines, NCI-H157, GLC-82, Calu-3, LTEP-s, NCI-H520, NCI-H209, and PG-LH7 were cultured and the expression of RNase L was evaluated by ICC and WB. As the results showed, RNase L was highly elevated in all lung cancer cell lines but not in breast cancer cell line (served as a positive control) (Fig. 1a). As RNase L functions in the cytoplasm as endoribonuclease to catalyze ssRNA or rRNA, previous research mainly focused on its cytoplasmic function^22^. However, as the results showed in Fig. 1a, the nuclear localization of RNase L was also observed. Therefore, we further measured the expression in specific fractions, respectively, and found that RNase L was elevated in lung cancer cells (Fig. 1b) not only in cytoplasm (Fig. 1c) but also in nucleus (Fig. 1d), indicating the different function of RNase L in lung cancer cells.

After we proved the high expression of RNase L in lung cancer cells, we asked whether the elevated RNase L showed elevated catalytic activity accordingly. 2–5A, a specific activator of RNase L^23^, was applied in lung epithelial cell, BEAS-2B, to analyze the time course of cleavage activity of RNase L. We found that the application of 2–5A for 24 h showed the most active cleavage to 28 s rRNA (Fig. 1e). Adopting this verified time of 2–5A application, we measured the cleavage activity of RNase L in lung cancer cell lines and found that the cleavage activity was suppressed, in varying degrees, in all lung cancer cells, especially in lung adenocarcinoma cell line, NCI-H157 (Fig. 1f). Thus these results indicated that the expression of RNase L is elevated while the catalytic activity is suppressed in lung cancer cells.

### The nuclear function of RNase L is suppressed in lung cancer cells

**Fig. 2:**
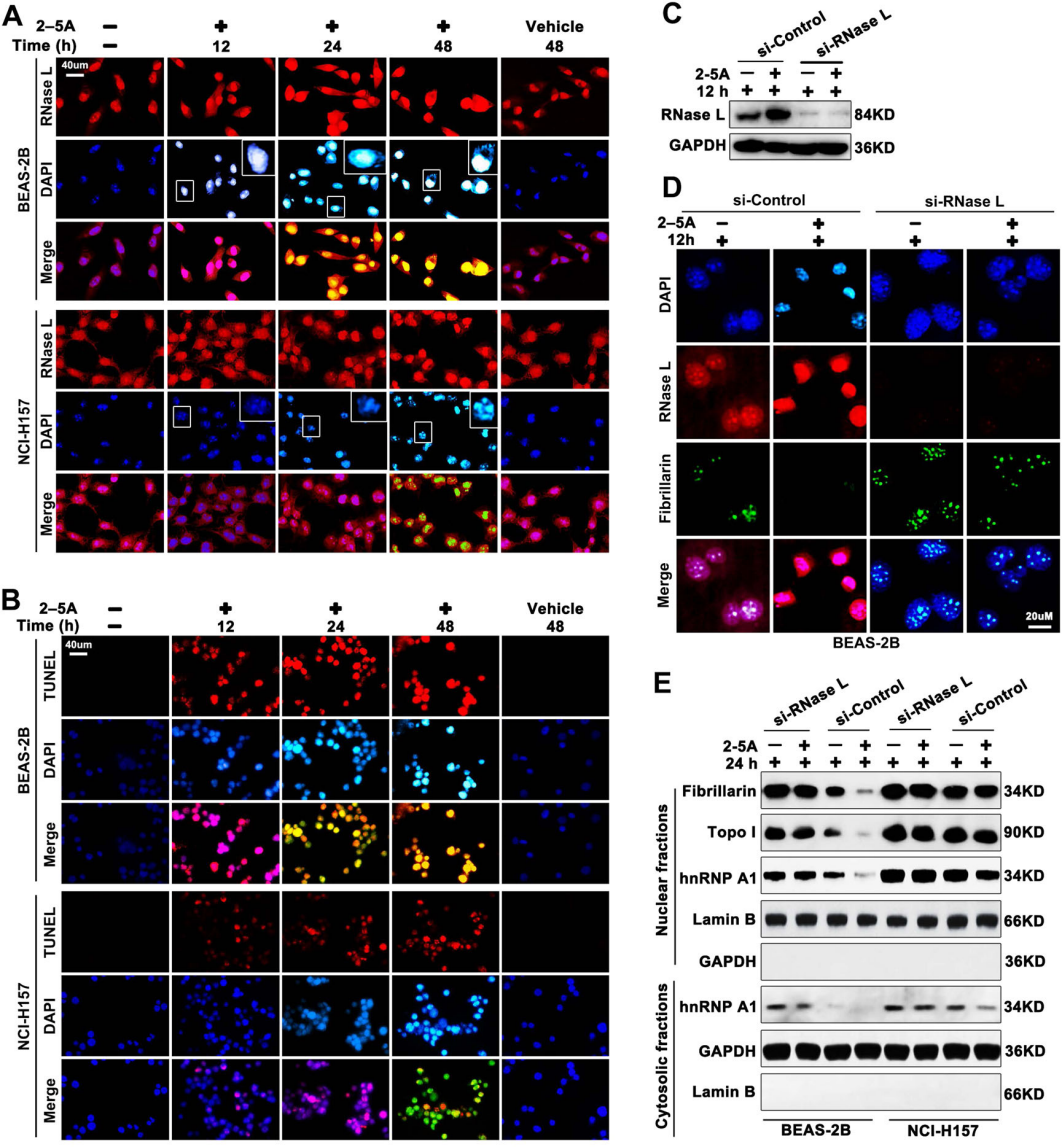
RNase L functions in the nucleus and induces nuclear condensation and DNA cleavage in lung epithelial cells. Immunocytofluorescence of RNase L and DAPI in BEAS-2B and NCI-H157 stimulated by 2–5A at the indicated time points. **b** Immunocytofluorescence of TUNEL and DAPI in BEAS-2B and NCI-H157 stimulated by 2–5A at the indicated time points. **c** Western blot of RNase L in BEAS-2B interfered with si-RNase L or not. **d** Immunocytofluorescence of RNase L, DAPI, and Fibrillarin in BEAS-2B interfered with si-RNase L or not. **e** Western blot of fibrillarin, Topo I, and hnRNP A1 in the nucleus or cytoplasmic fractions; Lamin B and GAPDH are regarded as the internal reference of *n* nucleus and cytoplasmic fractions, respectively

Except for the suppressed cytoplasmic function of RNase L, we wondered whether the function of RNase L that was highly expressed was also impaired. To answer this question, the nuclear function of RNase L should be elucidated. Previous research proposed the apoptosis-inducing function of RNase L^20,24^, and we asked whether the apoptosis is related to the function of RNase L in the nucleus. Through ICF in cultured cells, we found that RNase L could induce the condensation of the nucleus in BEAS-2B, starting from 12 h in the nucleolus and peaked at 48 h after the application of 2–5A. However, though 2–5A also elevated the expression of RNase L in NCI-H157 cells, the nuclear condensation, indicated by DAPI, was not initiated from nucleolus as BEAS-2B showed suppressed and heterogeneous condensation throughout the nucleus (Fig. 2a). Nuclear condensation usually accompanied with DNA cleavage and marked by TUNEL^25^. Therefore, we further analyzed whether RNase L could also induce DNA cleavage. As the results showed, DNA cleavage was synchronously occurred with nuclear condensation after 2–5A stimulation in BEAS-2B cells, while this phenomenon is suppressed and delayed in NCI-H157 that received the same stimulation (Fig. 2b).

As 2–5A stimulation in BEAS-2B cells leads to the absence of the granular component (GC), we speculate whether RNase L could function in the GC region. GC was the precursor of the ribosome subunits and Fibrillarin was reported to be located at GC region to participate in methylation of rRNA^25–27^. Therefore, we investigated the influence of RNase L to Fibrillarin. As the results showed, elevated RNase L expression accompanied with nuclear condensation and decreased Fibrillarin, while this phenomenon was not recurrent after RNase L interference (Fig. 2c, d). These results indicated the important function of RNase L in the nucleus that it induces condensation, DNA cleavage, and downregulation of Fibrillarin.

Fibrillarin was reported to participated rRNA transcription^26^, which binds to Topoisomerase I (Topo I) at N terminal that promotes rRNA transcription^28,29^. Topo I could further bind to hnRNP A1 at C terminal to exert functions like DNA endonuclease and ligase^28,29^. hnRNP A1 could further translocate to the cytoplasm to stabilize mRNA and promote translation^30,31^. Therefore, Fibrillarin, Topo I and hnRNP A1 interact together to promote the transcription of rRNA and mRNA and the translation of mRNA. On the contrary, RNase L could cleavage rRNA and viral mRNA. There is also a study reported that it downregulates RNA in mitochondria^32^. These results indicate an opposite role of RNase L to Fibrillarin, Topo I, and hnRNP A1 in mRNA and rRNA transcription as well as mRNA translation. Therefore, we analyzed the effects of RNase L activation to Fibrillarin, Topo I, and hnRNP A1. As the results showed, RNase L activation significantly downregulated the Fibrillarin expression (Fig. 2e) in BEAS-2B, which is in accordance with the result in Fig. 2d. Moreover, RNase L also decreased the Topo I expression (Fig. 2e). Decreased Topo I could suppress RNA transcription and induce the DNA breakage^33^, indicating that RNase L may induce nuclear condensation and DNA cleavage through the downregulation of Fibrillarin and Topo I. In addition, RNase L also decreased hnRNP A1 expression in the nucleus and cytoplasm, suggesting that hnRNP A1 may serve as an intermediary of RNase L in regulating downstream target genes. However, Topo I, Fibrillarin, and hnRNP A1 all remained at high levels in NCI-H157 cells after the 2–5A stimulation (Fig. 2e), indicating the impaired function of RNase L in lung cancer cells.

### RLI binds to RNase L and inhibits its function in lung cancer cells

**Fig. 3:**
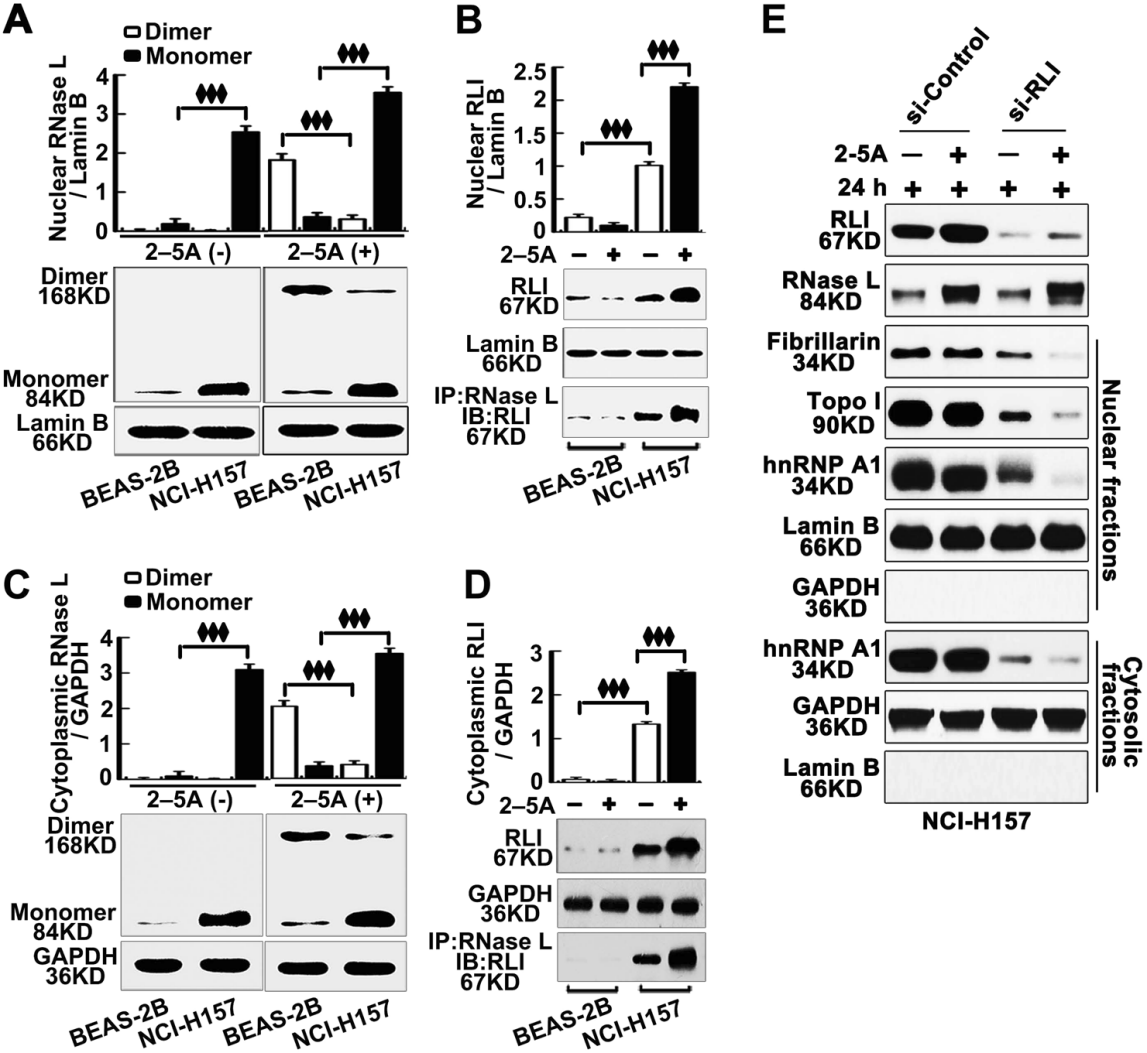
Suppressed dimerization of RNase L in lung cancer cells due to interaction with RLI. **a**, **c** Western blot of monomer and dimer of RNase L in the nucleus or cytoplasmic of BEAS-2B and NCI-H157 cells. **b**, **d** Western blot of RLI and IP of RNase L and RLI in the nucleus and cytoplasm in BEAS-2B and NCI-H157. **e** Western blot of RLI and RNase L in total cell lysate, fibrillarin, Topo I, and hnRNP A1 in the nucleus or cytosolic fractions after the interference of RLI (si-RLI)

Hence, we further asked why RNase L remains an impaired function in NCI-H157 cells, though expressed at a high level. Dimerization of RNase L is the basis for its function and RLI was reported to inhibit the function of RNase L^18,34,35^. We found that, both in cytoplasm and nucleus, dimerization of RNase L was significantly suppressed in NCI-H157 cells after 2–5A stimulation (Fig. 3a, c). Meanwhile, 2–5A stimulation also increased the level of RLI in NCI-H157 cells and interacted with RNase L to inhibit its function (Fig. 3b, d). Notably, further research found that, even in the absence of 2–5A stimulation, RLI in lung cancer cells still interacted with RNase L (Fig. 3b, d). The interaction between RNase L and RLI indicated that, though RNase L in lung cancer cells is upregulated, most of the RNase L interacted with RLI and the monomer of RNase L remains limited, leading to decreased dimerization of RNase L after 2–5A stimulation in lung cancer cells. Thus the dimerization of RNase L not only depends on the expression of RNase L but also relates to the expression of RLI and the interaction between RLI and RNase L. To prove this, we interfered RLI and analyzed the expression of Fibrillarin, Topo I, and hnRNP A1 after 2–5A stimulation. The results showed that Fibrillarin, Topo I, and hnRNP A1 were downregulated by RNase L activation after the RLI interference (Fig. 3e), indicating that RLI was the reason of impaired regulation of RNase L to these genes.

Thus these results proved that the elevated RLI in nucleus and cytoplasm in lung cancer cells, which interacts with RNase L that inhibits the dimerization, is the reason for the functional impairment of RNase L.

### IFN-γ restored RNase L function by enhancing its expression that neutralized RLI

Type I IFN has been reported to regulate the 2–5A/RNase L system in antiviral immunity and shows, at the same time, antitumor activity in multiple models^1,3,19^.

**Fig. 4:**
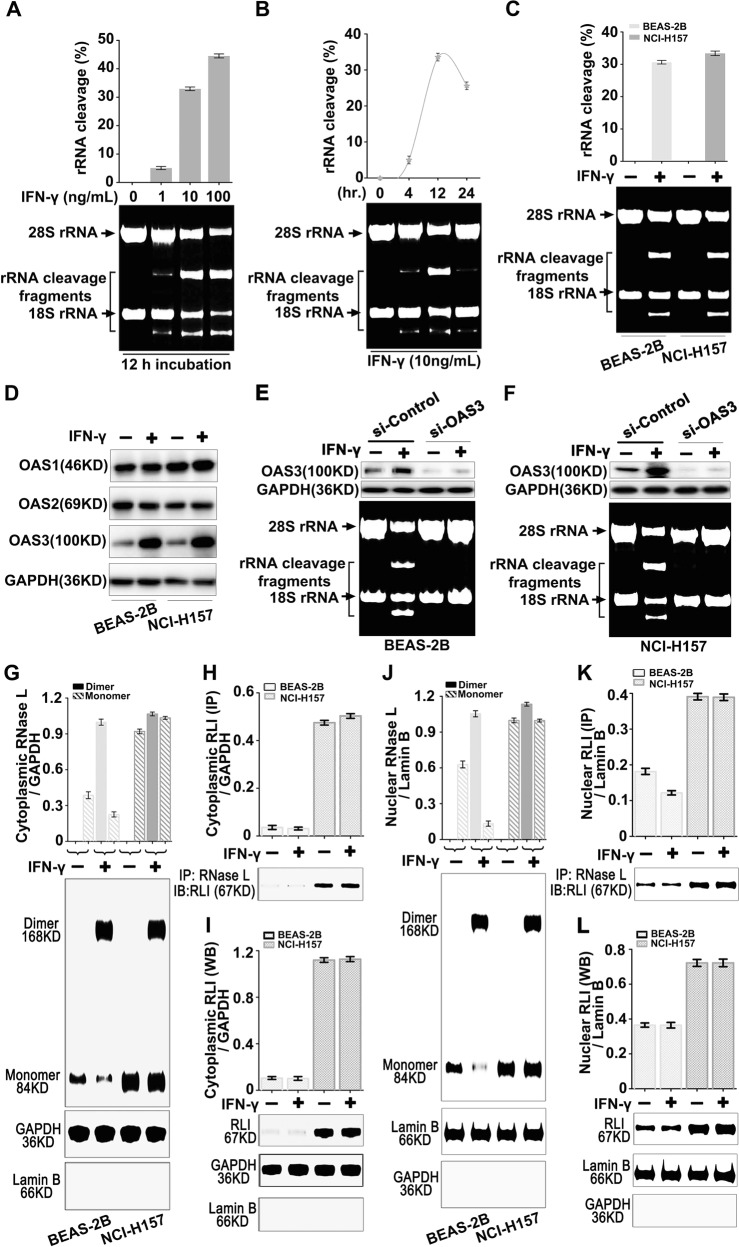
Upregulation of RNase L by IFN-γ neutralized RLI and restored cleavage activity. **a**, **b** Dose dependence (**a**) and time course (**b**) of IFN-γ in restoring the cleavage activity of RNase L (*n* = 3). **c** Cleavage activity of RNase L in the presence of IFN-γ in BEAS-2B and NCI-H157 cells (*n* = 3). **d** Western blot of OAS1, OAS2, and OAS3 in the presence of IFN-γ in BEAS-2B and NCI-H157 cells. **e**, **f** Western blot verification and rRNA cleavage after OAS3 interference by siRNA; **g**, **j** Western blot of RNase L dimer or monomer in the cytoplasm (**g**) or nucleus (**j**) in the presence of IFN-γ in BEAS-2B and NCI-H157 cells. **h**, **i**, **k**, **l** Cytoplasmic (**h**, **i**) and nuclear (**k**, **l**) RLI expression (**i**, **l**) and IP with RNase L (**h**, **k**) in the presence of IFN-γ in BEAS-2B and NCI-H157 cells

The IFN induces the expression of OAS, and the latter synthesize 2–5A from ATP and further activate RNase L^6,36^, indicating that IFN could activate RNase L indirectly. Therefore, we asked whether IFN-γ could activate RNase L. As the results showed, IFN-γ induced the cleavage activity of RNase L after 12-h treatment in a dose-dependent manner (Fig. 4a, b). And the cleavage activity after IFN-γ treatment in NCI-H157 showed similar level compared with BEAS-2B (Fig. 4c), suggesting the restored function of RNase L in lung cancer cells. Moreover, we found that IFN-γ elevated the expression of OAS3, rather than of OAS1 or OAS2 (Fig. 4d), and the knockdown of the OAS3 further suppressed the cleavage of rRNA (Fig. 4e, f), indicating that the activation of RNase L by IFN-γ was dependent on OAS3. Mechanistically, we found that IFN-γ induced the dimerization of RNase L (Fig. 4g, j) and inhibited the elevation of RLI (Fig. 4i, l), releasing the interaction of RLI and RNase L (Fig. 4h, k).

### IFN-γ promoted the RNase L-dependent mitochondria-mediated apoptosis in lung cancer cells

**Fig. 5:**
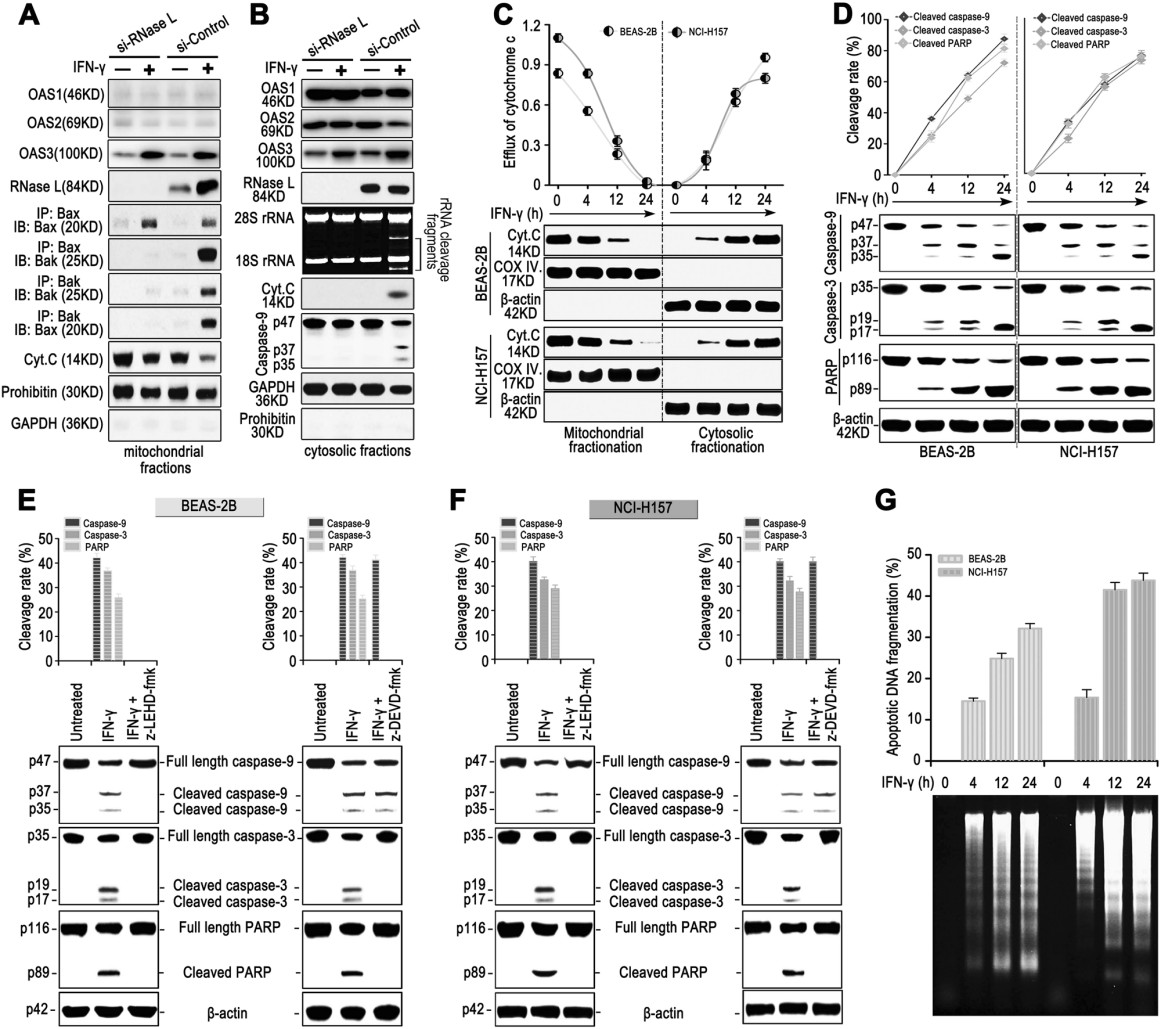
IFN-γ promoted apoptosis activated by RNase L. **a**, **b** Western blot of OAS1, OAS2, OAS3, RNase L, cytochrome C, and Caspase-9 in the mitochondrial or cytosolic fraction, co-immunoprecipitation of Bax and Bak, and cleavage activity of RNase L in NCl-H157 cells after the interference of RNase L. Prohibitin and GAPDH were regarded as the internal reference of mitochondria or cytosolic fractions, respectively. **c** Mitochondrial or cytosolic efflux of cytochrome C at different time points of IFN-γ stimulation (*n* = 3). **d** Western blot of Caspase-9, Caspase-3, and PARP in BEAS-2B and NCI-H157 cells stimulated with IFN-γ at different time points (*n* = 3). **e**, **f** Western blot of Caspase-9, Caspase-3, and PARP in BEAS-2B (**e**) and NCl-H157 (f) cells stimulated with IFN-γ and Caspase-9 inhibitor (z-LEHD-fmk) or Caspase-3 inhibitor (z-DEVD-fmk) (*n* = 3). **g** Accumulated DNA ladder after IFN-γ stimulation in BEAS-2B and NCI-H157 cells (*n* = 3)

Results above reveal that the activation of RNase L by IFN-γ cleave rRNA and further induce apoptosis as a signal^10^. Previous research reported that the IFN-α-induced activation of RNase L could suppress protein translation in mitochondria^24^. Another research also discovered that RNase L from mitochondria in Hela cells could be activated by IFN-α and cleave mRNA in mitochondria, including mRNA encoding cytochrome b, ATPase 6, and cytochrome oxidase subunit II^18^. These results indicated that IFN could promote activation and function of RNase L in mitochondria. We wondered whether IFN-γ could induce the activation of RNase L and promote mitochondria apoptosis. As the results showed, we found that OAS3 located at mitochondria could be elevated after IFN-γ treatment. Meanwhile, RNase L was also recruited to mitochondria by IFN-γ (Fig. 5a) and promoted the formation of the heterodimer of Bax and Bak as well as the homodimer of Bak (Fig. 5a), leading to the release of cytochrome C from mitochondria to cytoplasm (Fig. 5a, b) and the activation of Caspase-9 (Fig. 5b). Moreover, we found that OAS3 located at cytoplasm could be elevated by IFN-γ, activate RNase L, and promote the cleavage of rRNA. Therefore, these results indicated that the activation of RNase L by IFN-γ could initiate the rRNA cleavage in cytoplasm and apoptosis through mitochondria.

We further asked whether the mitochondria-initiated apoptosis induced by RNase L could be restored in lung cancer cells. We found that IFN-γ-induced apoptosis in lung cancer cells shared the similar apoptotic process as BEAS-2B, including the release of cytochrome C and the activation of Caspase-9, Caspase-3, and PARP (Fig. 5c, d). Through applying specific inhibitor of Caspase-9 (z-LEHD-fmk) and Caspase-3 (z-DEVD-fmk), we further proved that apoptosis activation was dependent on Caspase-9→Caspase-3→PARP signaling (Fig. 5e, f). The cleavage of PARP exposed the nucleosome that could easily be cleaved by Caspase-activated endonuclease^9^. To prove this phenomenon, we extracted the DNA of BEAS-2B and NCI-H157 cells after IFN-γ treatment and found the accumulation of apoptotic DNA ladder (Fig. 5g), indicating that IFN-γ promoted the RNase L-dependent mitochondria-mediated apoptotic signaling and led to apoptosis in the nucleus.

**Fig. 6:**
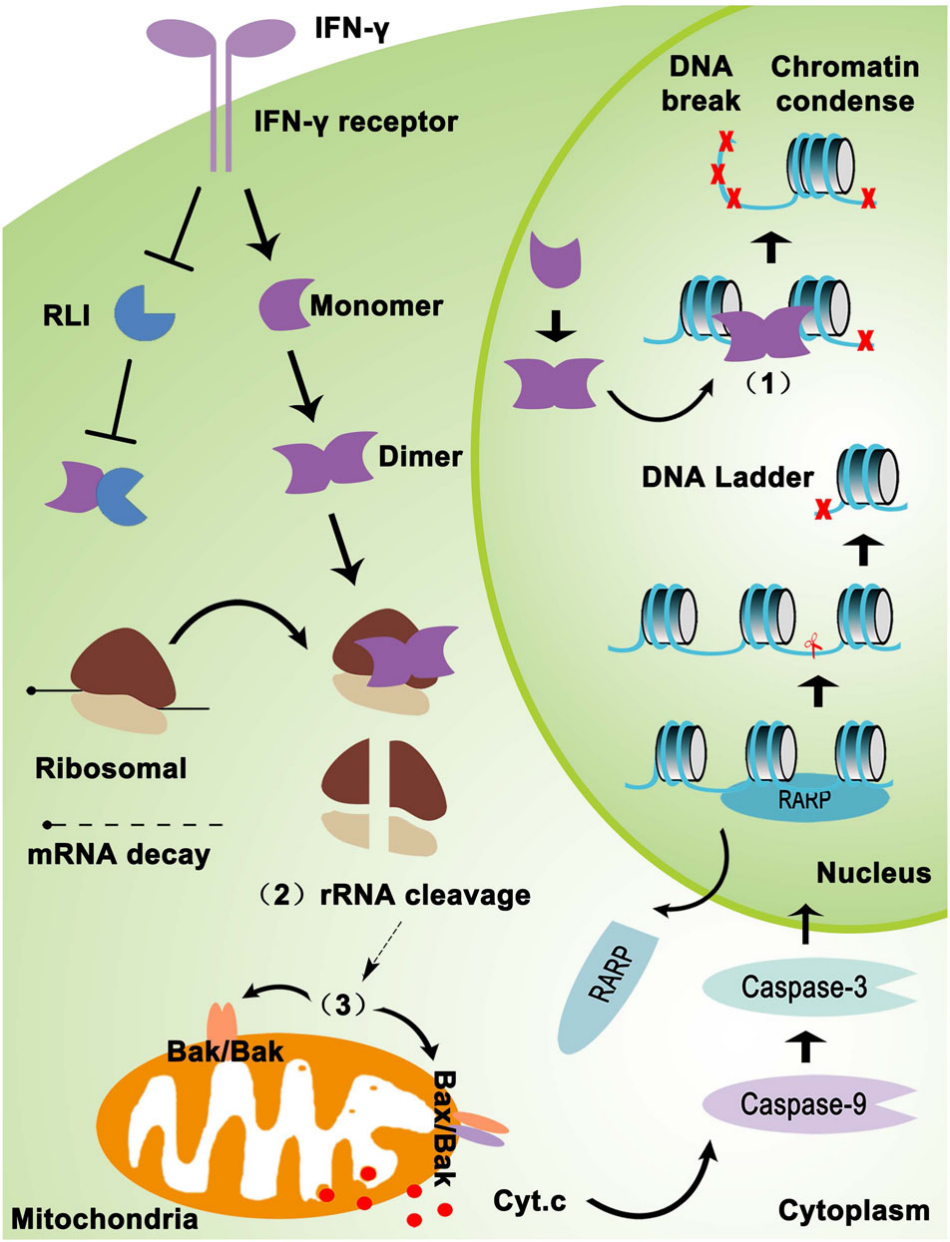
**Graphical abstract of the mechanism of IFN-γ promoting the RNase L function**

## Discussion

Investigating molecules with abnormal expression and function in cancer cells helps to understand the mechanisms of the cancerous phenotype^14^. In the present study, we reported that RNase L, an endoribonuclease of 2–5A/RNase L system in IFN-mediated antiviral signaling, is upregulated with impaired function in lung cancer cells due to altered coordination between RNase L and its inhibitor, RLI. In normal lung epithelial cells, treatment of 2–5A induces the expression of RNase L but did not influence the expression of RLI. This leads to an increased level of RNase L homodimer with activated functions and further apoptosis induction. However, in lung cancer cells, the elevation of RNase L expression was companied by increasing RLI. RLI interacts with RNase L and inhibits its activation, sustaining cancer cell survival (Supplemental Fig. 1). Previous research reported that impaired function of RNase L in cancer mainly resulted from mutation and thus led to cancer progression^37^. However, in the present study, we find that RNase L with the impaired function was due to the elevated expression of RLI. Therefore, our finding provides new insight into how cancer cells escape the antiproliferative mechanism in the host to form a tumor, which may be useful for the design of novel strategies for treating lung cancer through regulating the coordination of RNase L/RLI.

Several studies have reported the antitumor effects of RNase L through several mechanisms, including the dependence on enzyme activity and regulations to specific signaling^3,14,37,38^. However, these studies mainly investigated the expression feature of RNase L and subsequent alteration of oncogenes that participated in cancer progression. It remains largely unknown about how RNase L altered functionally or mechanistically when compared with normal cells. In the present study, by comparing lung cancer cells and normal pulmonary epithelial cells, we investigated the difference and altered phenotype that related to the function of RNase L between two kinds of cells and proposed the mechanism of how cancer cells impaired the function of RNase L. Another highlight of the present study is the function of RNase L in the nucleus. Previous studies focused on the function of RNase L in the cytoplasm that regulates signaling or molecules involved in cancer progression^3,14,38^. In our research, we found that RNase L was also located at the nucleus with high expression level both in lung epithelial cells and cancer cells. In the nucleus, RNase L significantly promoted the nuclear condensation in lung epithelial cells but was impaired in cancer cells. Notably, condensation in epithelial cells was started from the GC. While in lung cancer cells, GC was still maintained after 2–5A treatment (Fig. 2a). GC is the precursor of the ribosome subunit, and the region where GC located is responsible for the processing of the precursor rRNA and the assembly of the ribosome subunit^25,39^. As a nucleolin in GC, Fibrillarin participated in the methylation of rRNA^27^ and the assembly of the subunit of ribosome^26^. Research has reported that Fibrillarin is elevated in multiple cancer cells and lead to the altered expression pattern of nucleolin, promoting the expression of oncogenes^17^. In the present study, we found RNase L induced the downregulation of Fibrillarin and nuclear condensation, whereas in lung cancer cells, elevated RLI inhibits the function of RNase L and suppresses the apoptosis. Thus the present study highlighted an important mechanism in lung cancer cells that protected the cells from the nucleolus-inhibiting effects and induction of apoptosis through impairing RNase L.

Except for nuclear condensation, RNase L also promotes DNA cleavage that was shown by TUNEL. This process was further proved by RNase L suppressing the Topo I expression. Previous research has reported that Topo I promotes several rRNAs and mRNA transcription that are associated with cancerous phenotype and its downregulation induces DNA cleavage and apoptosis^28,29,33^. Unsuppressed Topo I could bind to fibrillarin at N terminal that promotes rRNA transcription or bind to hnRNP A1 at C terminal to exert functions like DNA endonuclease and ligase^28,29^. hnRNP A1 could further translocate to the cytoplasm to stabilize mRNA and promote translation^30,31^. Hence, RNase L-mediated Topo I and hnRNP A1 suppression may lead to transcriptional and translational inhibition and cell apoptosis. Therefore, the present study proposed an important mechanism of RNase L in antitumor effects and oncogene regulations.

RNase L-mediated transcriptional inhibition and apoptosis initiation in cancer cells was impaired by the elevated expression of RLI, leading to cell survival and progression of cancer cells. RLI was reported to be upregulated in several cancer cells^3^ and 2–5A treatment in NCI-H157 cells led to a synchronous elevation of both RNase L and RLI, which still impaired the function of RNase L and subsequent apoptosis. This also indicates that targeting RLI to restore the function of RNase L may be an option for lung cancer with RNase L functional deficiency.

In restoring the function of RNase L, we found that application of IFN-γ promoted the RNase L function but did not increase the expression of RLI (Supplemental Fig. 1). Thus, though IFN-γ did not influence the expression of RLI, IFN-γ-mediated upregulation of RNase L neutralized the inhibitory effects of RLI and maintained a relatively high level of the homodimers of RNase L, suggesting possible therapeutic efficacy for RNase L-positive lung cancer. Previous studies unveiled that IFN-based immunotherapy acts as a double-edged sword for cancer treatment^1,2,19^. On the one hand, IFN induces T helper type 1 polarization, cytotoxic T lymphocyte activation, and dendritic cell tumoricidal activity that promotes antitumor immunity and suppress cancer progression^1^^[,19^. On the other hand, IFN also enhances the expression of programmed death ligand 1 in cancer cells that contributes to immune-escape mechanism^2^. Unlike these researches that focused on the interaction of immunity and cancer cells, the present study unveiled a unique mechanism that IFN-γ promotes the apoptosis of cancer cell dependent on RNase L. The IFN-γ application promotes the homodimer formation of RNase L. Activated RNase L may further catalyze the subunits of ribosome and produces segments of rRNA, which, like a damage signal, initiate mitochondrial apoptosis. We found that IFN-γ induced the formation of Bax-Bak heterodimer and Bak homodimer in mitochondria. Meanwhile, cytochrome C was released into the cytoplasm and activated Caspase-9–Caspase-3–PARP cascades, eventually activating mitochondria-mediated apoptosis (Fig. 6). Therefore, IFN-γ therapy combined with RLI inhibition may be a promising treatment for lung cancer.

Collectively, the present study investigated the expression, impaired function, and related mechanism of RNase L in lung cancer cells. The application of IFN-γ restored the RNase L function through elevating its expression that neutralizes the inhibitory effects and promotes RNase L-dependent mitochondrial-mediated apoptosis in lung cancer cells. The present study highlighted a unique mechanism of IFN-γ in cancer suppression and indicated a possible application as an immune adjuvant for future cancer immunotherapy.

## Supplementary information

Supplemental Figure 1

supplementary figure legends
