# Supplementary figures and images for: IFN-γ restores the impaired function of RNase L and induces mitochondria-mediated apoptosis in lung cancer
Source: Cell Death Dis. 2019 Sep 9;10(9):642. doi: 10.1038/s41419-019-1902-9 (PMC6733796; doi:10.1038/s41419-019-1902-9)

A

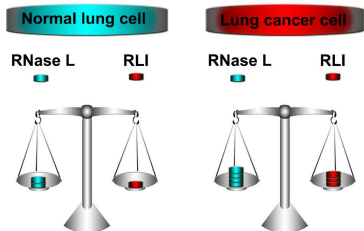

B

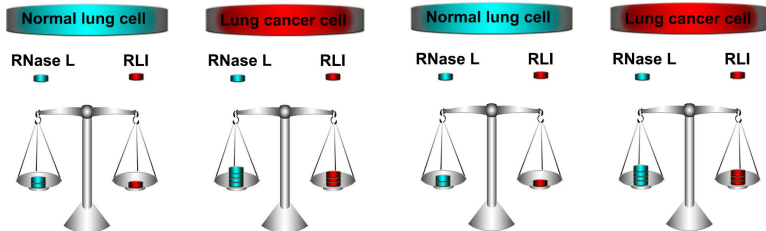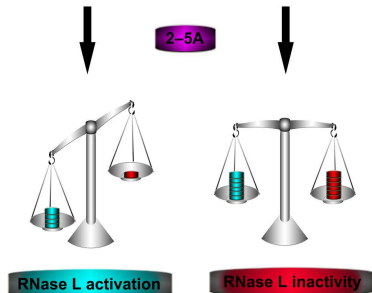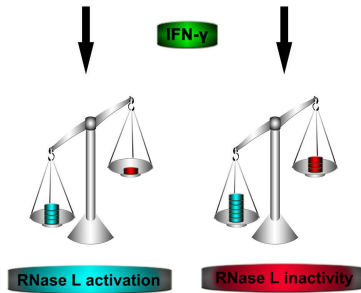

Supplement: Supplementary file 1 — Supplemental Figure 1 [file 41419_2019_1902_MOESM1_ESM.pdf]
