# Supplementary material for: IFN-γ restores the impaired function of RNase L and induces mitochondria-mediated apoptosis in lung cancer
Source: Cell Death Dis. 2019 Sep 9;10(9):642. doi: 10.1038/s41419-019-1902-9 (PMC6733796; doi:10.1038/s41419-019-1902-9)
Supplement: Supplementary file 2 — supplementary figure legends [file 41419_2019_1902_MOESM2_ESM.docx]

**Supplemental Figure 1. Interpretation of IFN-γ restoring RNase L function in lung cancer cells.**

A: In normal lung epithelial cells, 2-5A stimulation enhanced the expression of RNase L but didn’t alter RLI, leading to the dimerization and activation of RNase L. While in lung cancer cells, 2-5A stimulation leads to synchronous elevation of RNase L and RLI, resulting impairment of RNase L function. B: IFN-γ enhanced the expression of RNase L but didn’t alter RLI expression in lung cancer cells. Thus, RNase L outnumbered RLI and dimerized that restored the function of RNase L.
